# Supplementary material for: Replication of Resting State-Task Network Correspondence and Novel Findings on Brain Network Activation During Task fMRI in the Human Connectome Project Study
Source: Sci Rep. 2018 Dec 3;8:17543. doi: 10.1038/s41598-018-35209-6 (PMC6277426; doi:10.1038/s41598-018-35209-6)
Supplement: Supplementary file 1 — Supplemental Information [file 41598_2018_35209_MOESM1_ESM.docx]

Replication of Resting State-Task Network Correspondence and Novel Findings on Brain Network Activation During Task fMRI in the Human Connectome Project Study

Lisa D. Nickerson*^,1,2^

^1^ Applied Neuroimaging Statistics Lab, McLean Hospital, Belmont, MA, USA

^2^ Department of Psychiatry, Harvard Medical School, Harvard University, Boston, MA, USA

**Correspondence should be addressed to:** LN ([lisa_nickerson@hms.harvard.edu](mailto:lisa_nickerson@hms.harvard.edu))

Table 1. Correspondence between D20 GICA spatial maps (HCP rest, HCP task, Smith rest, Smith BM). The first ten networks listed in the table are the ten networks with high correspondence between Smith Rest and Smith BrainMap that were shown in Smith Figure 1. Other networks identified in each dataset are also listed. Networks that appeared to be split into multiple components are a mixture of networks is listed in parentheses.

| **Network** | **HCP Rest** | **HCP Task** | **Smith Rest** | **Smith BM** |
| --- | --- | --- | --- | --- |
| Medial Visual | 3 | 6 | 5 | 8 |
| Lateral Occipital 1 | 6 | 19 | 15 | 5 |
| Lateral Occipital 2 | 0 | 17 | 9 (mixed with DAN) | 16 |
| Default Mode Network (DMN) | 2 | 7 | 6 | 17 |
| Cerebellum | 14 | 4 | 8 | 7 |
| Sensorimotor | 7 | 13 | 1 | 6 |
| Auditory | 8 | 5 | 2 | 10 |
| Executive Control | 15 | 11 | 7 | split: 2,15,19 |
| Right Frontoparietal Network | 5 | 0 | 12 | 12 |
| Left Frontoparietal Network | 4 | 1 | 11 | 3 |
| Dorsal Attention Network (DAN) | 1 | 9 | 9 (mixed with  Lat Occ 2),  16 (mixed with DMN-related) | 1 |
| DMN-Related | 10 | 16 (mixed with VAN) | 14 | -- |
| DMN-Related | -- | 2 | 10 | -- |
| Ventral Frontal Cortex | 16 | 3 | 13 | 14 |
| Ventral Attention Network (VAN) | 9 | 16 (mixed with DMN) | -- | -- |
| Amygdala/Hippocampus/Brainstem | -- | -- | 3 | -- |
| Basal Ganglia | -- | -- | -- | 4 |
| Cingulo-Opercular Network | -- | -- | -- | 11 |
| Lateral Occipital 3 | -- | 12 | -- | -- |
| Lateral Occipital 4 | -- | 19 | -- | -- |
| Sensorimotor 2 | -- | 14 | -- | -- |
| Brainstem | -- | -- | 4 | -- |

Table 2. Correspondence between D70 GICA HCP resting state and task fMRI maps. Also shown are the spatial cross-correlation between the Smith maps shown for D70 in his Figure 3. -- indicates an unknown or sub-network that did not have a corresponding network in other results.

| **HCP Rest** | **HCP Task** | **Spatial CC (HCP Rest/Task)** | **Smith Figure 3 Map** | **HCP Rest # (Spatial CC**  **with Smith Map)** |
| --- | --- | --- | --- | --- |
| 0 | 13 | 0.52 | 3 | 17 (0.7) |
| 1 | 4 | 0.37 |  |  |
| 2 | 1 | 0.5 |  |  |
| 3 | 5 | 0.76 | 1 | 0 (.69) |
| 4 | 0 | 0.73 |  |  |
| 5 | 37 | 0.45 | 7 | 10 (0.51) |
| 6 | -- |  |  |  |
| 7 | 51 | 0.49 |  |  |
| 8 | artifact |  |  |  |
| 9 | artifact |  |  |  |
| 10 | artifact |  |  |  |
| 11 | 10 | 0.72 | 9 | 45 (0.62) |
| 12 | 23 | 0.53 |  |  |
| 13 | 8 | 0.85 |  |  |
| 14 | 28 | 0.29 |  |  |
| 15 | 25 | 0.61 |  |  |
| 16 | 19 | 0.58 |  |  |
| 17 | 54 | 0.46 | 5 | 1 (0.36) |
| 18 | 14 | 0.63 |  |  |
| 19 | 11 | 0.69 |  |  |
| 20 | 45 | 0.44 |  |  |
| 21 | 62 | 0.32 |  |  |
| 22 | 58 | 0.43 |  |  |
| 23 | artifact |  |  |  |
| 24 | 49 | 0.5 | 4 | 27 (0.37) |
| 25 | 18 | 0.57 |  |  |
| 26 | 16 | 0.38 |  |  |
| 27 | 2 | 0.63 |  |  |
| 28 | 55 | 0.48 | 6 | 24 (0.41) |
| 29 | 56 | 0.48 | 2 | 53 (0.57) |
| 30 | 39 | 0.5 |  |  |
| 31 | artifact |  |  |  |
| 32 | 41 | 0.46 |  |  |
| 33 | 53 | 0.31 | 8 | 51 (0.44) |
| 34 | artifact |  |  |  |
| 35 | -- |  |  |  |
| 36 | 42 | 0.42 |  |  |
| 37 | 9 | 0.51 |  |  |
| 38 | artifact |  |  |  |
| 39 | artifact |  |  |  |
| 40 | 30 | 0.36 | 10 | 50 (0.45) |
| 41 | artifact |  |  |  |
| 42 | 15 | 0.42 |  |  |
| 43 | 4 | 0.37 |  |  |
| 44 | artifact |  |  |  |
| 45 | artifact |  |  |  |
| 46 | artifact |  |  |  |
| 47 | 21 | 0.44 |  |  |
| 48 | artifact |  |  |  |
| 49 | artifact |  |  |  |
| 50 | artifact |  |  |  |
| 51 | artifact |  |  |  |
| 52 | artifact |  |  |  |
| 53 | also 21 | 0.46 |  |  |
| 54 | 2 | 0.49 |  |  |
| 55 | artifact |  |  |  |
| 56 | artifact |  |  |  |
| 57 | artifact |  |  |  |
| 58 | artifact |  |  |  |
| 59 | artifact |  |  |  |
| 60 | artifact |  |  |  |
| 61 | artifact |  |  |  |
| 62 | 33 | 0.46 |  |  |
| 63 | artifact |  |  |  |
| 64 | artifact |  |  |  |
| 65 | artifact |  |  |  |
| 66 | artifact |  |  |  |
| 67 | artifact |  |  |  |
| 68 | artifact |  |  |  |
| 69 | artifact |  |  |  |
